# Endodontic Management of Open Apex Teeth Using Lyophilized Collagen Sponge and MTA Cement: Report of Two Cases 

**DOI:** 10.22037/iej.2017.48

**Published:** 2017

**Authors:** Miriam Graziele Magro, Milton Carlos Kuga, Weber Adad Ricci, Kátia Cristina Keine, Mateus Rodrigues Tonetto, Suellen Linares Lima, Alvaro Henrique Borges, Lauriê Garcia Belizário, Matheus Coêlho Bandeca

**Affiliations:** a * Department of Restorative Dentistry, Araraquara Dental School, Univ. Estadual Paulista, Araraquara, SP, Brazil;*; b *Department of Postgraduate Program in Integrated Dental Science, University of Cuiaba-UNIC, Cuiabá, MT, Brazil; *; c *Department of Postgraduate Program in Dentistry, CEUMA University-UNICEUMA, São Luis, MA, Brazil*

**Keywords:** Apex, Collagen, Endodontics, Mineral Trioxide Aggregate

## Abstract

Teeth with open apices, such as in immature teeth or those with apical root resorption are clinical cases with difficult immediate resolution. With the use of mineral trioxide aggregate (MTA) in dentistry, it was possible to optimize the treatment time of these cases by immediate placement of apical plug and the root canal filling. However, some negative effects can occur if MTA is extruded beyond the apex. To avoid this accident, it has been recommended to use of an apical matrix prior to placement of MTA. This study reports two clinical cases of apical plug placement in teeth with pulp necrosis and open apices. One case had an immature apex due to dental trauma and the other case had apical resorption due to the presence of endodontic infection in the root canal. MTA apical plug with approximately 4 mm thickness, was placed in the apical zone of the root and immediately the canal was obturated with gutta-percha and endodontic sealer. Follow-up evaluations showed clinical and radiographic evidence of success.

## Introduction

Open apices are a problem for the realization of the root canal treatment because they favor the extravasation of irrigating solution and/or sealer into periradicular tissues, which can have a negative effect on the apical healing process [1]. The main etiological factors for this occurrence are immature apexes of early-necrotized teeth or inflammatory apical root resorption [2, 3].

Therefore, to allow safe root canal filling, some techniques have been recommended, such as chemical or thermal adaptation of the gutta-percha in the radicular apical third and/or apexification with long-term intracanal calcium hydroxide dressing [3, 4]. However, these methods have many technical problems and require multiples treatment clinical sessions [3].

Apical adaptation of gutta-percha using heat or chemical agents, such as xylene or chloroform, do not provide adequate modeling of the root canal, and leaves spaces between the dentinal wall and gutta-percha. This facilitates the over extrusion of root canal sealer beyond the apex and/or apical microbial infiltration [4]. In addition, these chemical substances are potentially irritating and cytotoxic to periradicular tissue [5].

Although intracanal dressing of calcium hydroxide, is recommended for treatment of these cases, its intracanal use for a long time may reduce the resistance of root walls to fracture in future [3, 6, 7]. Some other problems include multiple treatment sessions and the risk of root canal contamination by microbial coronal leakage and the possibility of irregular shape of apical foramen and porous apical barrier [3, 8, 9].

**Figure 1:**
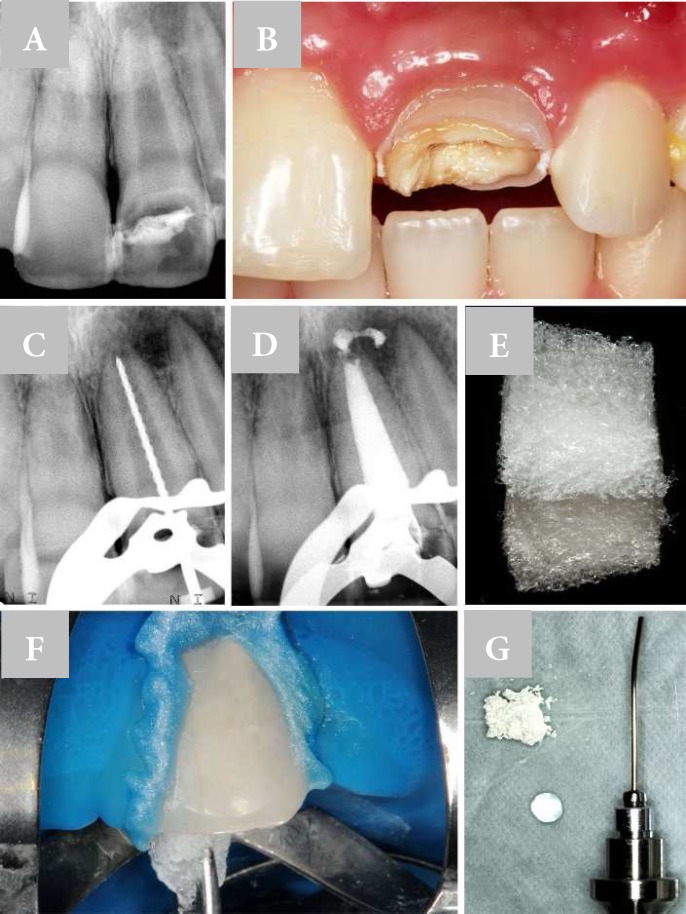
A)Open-apex immature tooth and presence of apical periodontitis; B) Horizontal dental crown fracture; C) Working length determination; D) Calcium hydroxide dressing; E) Lyophilized collagen sponge; F) Insertion of lyophilized collagen sponge in root canal; G) Device for insertion of MTA apical plug

In order to treat teeth with open apices in a short time, and to avoid the possible negative effects presented by prolonged use of intracanal calcium hydroxide medication, placement of an apical with freeze-dried bone, tricalcium phosphate, dehydrated dentin matrix or more recently, calcium silicate-based cements (CSC), such as mineral trioxide aggregate (MTA) and Biodentine, has been proposed [10-14].

MTA has good biological and physicochemical properties, and is one of the most recommended CSCs for use as apical barrier (apical plug) [15, 16]. However, it presents some disadvantages, mainly low resistance to solubilization due to their long final setting time and causing intense local inflammatory reaction if accidentally pushed into the apical periodontal tissues [2].

However, in order to avoid these complications, it has been proposed to use an additional apical matrix with collagen membrane, prior to placement of the apical barrier with MTA [13, 14]. However, the use of collagen membrane has some disadvantages, such high cost and the difficulty in handling the material. Lyophilized collagen sponge is a practical alternative, efficient and low cost, that is routinely recommended for promoting hemostasis at a surgical alveolus, besides its good biological properties [17, 18]. 

Therefore, this report presents two open apex teeth treated with lyophilized collagen sponge apical stop and MTA apical barrier prior to root canal filling.

## Case Report

***Case 1:*** A 12-year-old, male patient requested dental treatment of the upper left lateral incisor, two years after dental trauma due to car accident, with severe mobility. The tooth had initially received coronal access, but the patient did not catch up on the rest of the treatment.

A periapical radiography was taken which revealed the presence of a transversal radiolucent line, indicative of fracture in the dental crown, root apex with incomplete formation and periradicular radiolucent lesion (Figure 1A). Clinical findings verified the horizontal fracture of the middle third of the dental crown in the mesial-distal direction, with separation of incisor third fragment and exposure of the root canal (Figure 1B). There was no spontaneous pain and/or positive responses to thermal and electrical testing.

The incisal fragment was attached to the cervical segment using an adhesive system (Adper Scothbond; 3M, Sumaré, SP, BR) and composite resin (Z100; 3M, St. Paul, MN, USA). After finishing the coronal access , the root canal was filled with 3% sodium hypochlorite (ChlorCid V; Ultradent, South Jordan, Utah, USA) and a #80 K-file (Dentsply Maillefer, Ballaigues, Switzerland) was inserted 2 mm short of the estimated radiographic initial image, to obtain instrumentation length (Figure 1C). Upon completion of the chemo-mechanical preparation, irrigation was performed with 10 mL of saline, and as a final irrigation protocol, the root canal was flushed with 5 mL of 17% EDTA for 3 min, and finished with 10 mL of 3% sodium hypochlorite gel and 10 mL of saline, which was later aspirated and dried with absorbent paper points.

In sequence, calcium hydroxide intracanal dressing (Ultracal; Ultradent, South Jordan, Utah, USA) was maintained, for 72 h (Figure 1D). After this period, a new sequence of irrigation was performed with 5 mL of 17% EDTA, 10 mL of 3% sodium hypochlorite gel and 10 mL of saline. After drying the root canal, a fragment of hemostatic lyophilized collagen sponge (Hemospon; Technew, Rio de Janeiro, RJ, BR) was introduced and adapted in the apical third of the root canal with the aid of a #3 gutta-percha condenser (Figures 1E and 1F).

**Figure 2:**
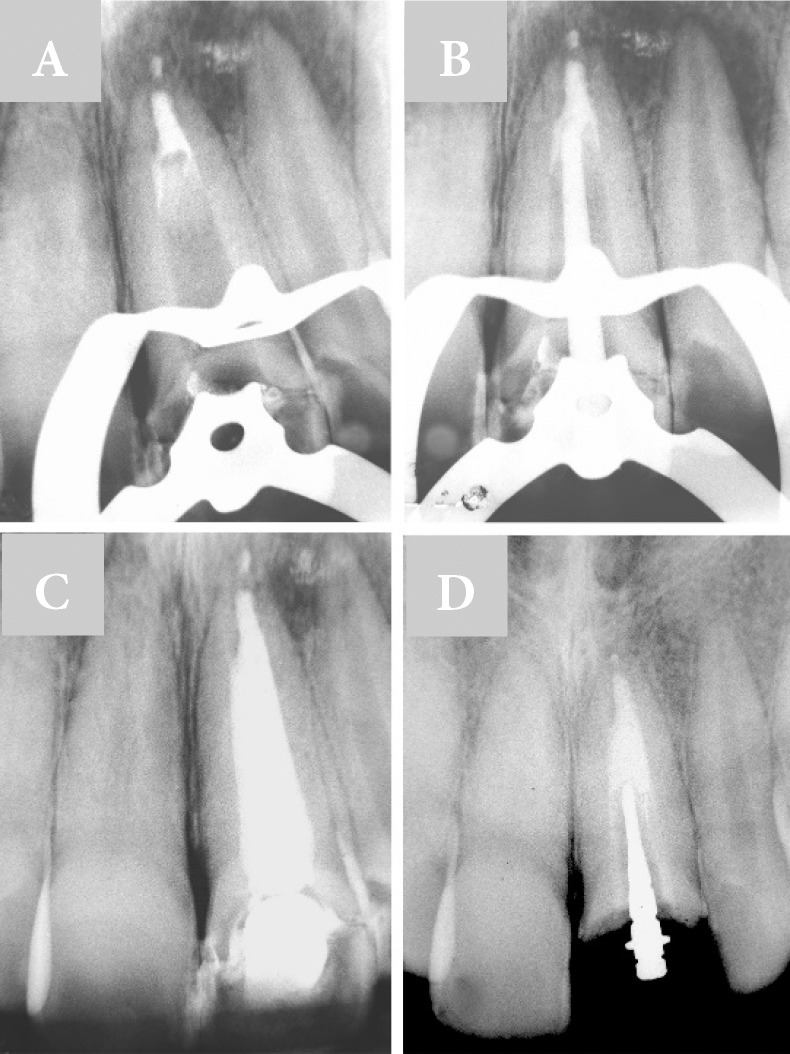
A) Lyophilized collagen sponge and MTA cement apical barrier; B) Apical barrier checking with gutta-percha point; C) Immediate root canal filling with calcium silicate-based sealer; D) Radiographic image after18 months

Immediately after compaction of the lyophilized collagen sponge, MTA (Angelus, Londrina, PR, BR) was mixed according to the manufacturer instructions and apical barrier was placed, in approximately 4 mm thickness in the apical third of the root canal with a special device for inserting material (Golgran , Sao Caetano do Sul, SP, BR) (Figure 1G). A new radiography was taken to verify the homogeneity of the apical plug (Figure 2A).

A #80 gutta-percha point was used for confirmation of the apical barrier (Figure 2B). The root canal was filled with gutta-percha points and calcium silicate-based sealer (MTA Fillapex; Angelus, Londrina, SP, BR), by lateral condensation technique. The coronal access was provisionally restored with glass ionomer cement (Vidreon; SS White, Rio de Janeiro, RJ, BR) (Figure 2C). The patient was instructed to follow the restorative procedures.

After 18 months, a new clinical and radiographic control was performed. There were no clinical signs of abnormality in the alveolar mucosa, and the treated tooth showed no sensitivity in vertical and or horizontal percussion. The radiographic assessment indicated local anatomical normality and total regression of the initial radiolucent lesion (Figure 2D).

**Figure 3:**
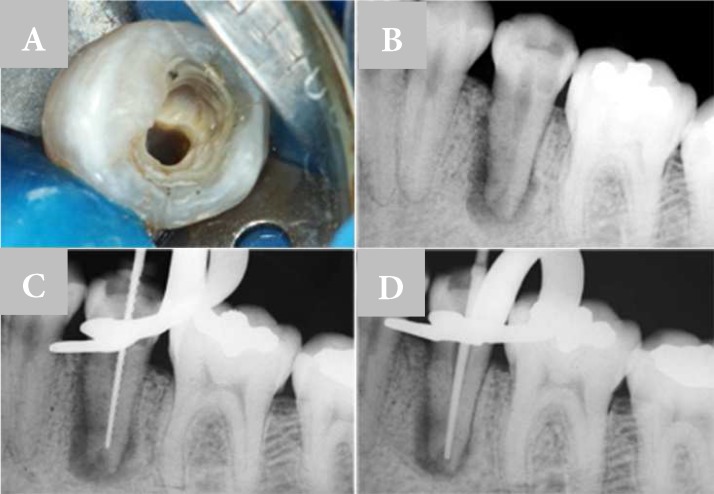
A) Coronal access; B) Apical periodontitis and apical root resorption; C) Working length determination; D) Checking working length with gutta-percha point

***Case 2:*** A male in his early thirties applied for dental treatment in the mandibular left second premolar with a report of previous periradicular abscess and emergency treatment. On examination absence of occlusal restoration was observed and coronal access had been previously prepared (Figure 3A). No clinical signs of edema and/or fistula in the alveolar mucosa was present. In radiographic analysis a periradicular radiolucent lesion surrounding a widely open apex and apical root resorption was evident (Figure 3B).

Initially, the root canal was irrigated with 10 mL of 3% sodium hypochlorite (V ChlorCid; Ultradent, South Jordan, Utah, USA), and cervical and middle thirds were prepared using rotary ProTaper instruments (Dentsply Maillefer, Ballaigues, Switzerland) up to S2 file. To determine the working length a #70 K-file was inserted in the root canal and a new radiographic image was obtained (Figure 3C). The root canal was instrumented 2 mm below the root end up to F5 instrument (ProTaper; Dentsply Maillefer, Ballaigues, Switzerland). Between each change of instrument, the root canal was filled with 5 mL of 3% sodium hypochlorite gel.

The final irrigation was performed with 5 mL of 17% EDTA (Biodinamica, Ibiporã, PR, BR) and 10 mL of 3% sodium hypochlorite gel, using insert E1 ultrasonic device (Helse, Ribeirão Preto, SP, BR) for 60s installed on an ultrasound unit (II Various; NSK, Shinagawa TKY, JP). After drying the root canal with absorbent paper points, F5 gutta-percha point (ProTaper; Dentsply Maillefer, Ballaigues, Switzerland) was used as the master cone. However, apical adaptation of the gutta-percha point was not possible and the procedure of MTA apical barrier placement after lyophilized collagen sponge was chosen (Figure 3D).

**Figure 4:**
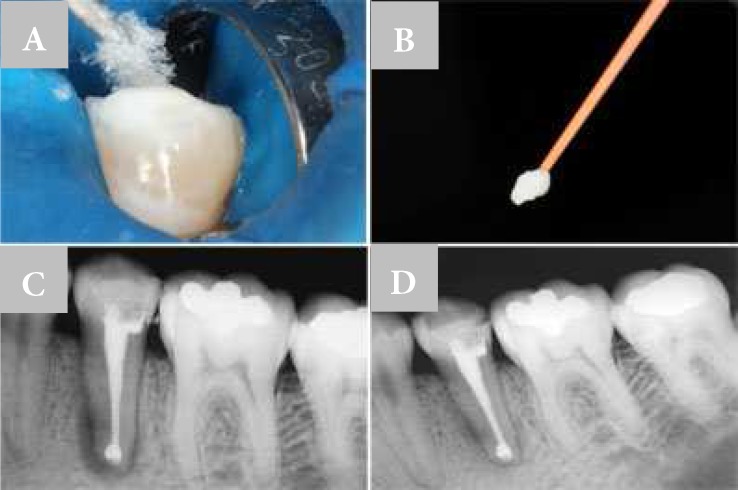
A) Insertion of lyophilized collagen sponge in the root canal; B) MTA cement insertion method; C) Immediate root canal filling with apical barrier; D) Six-month follow-up radiography

The lyophilized collagen sponge was inserted into the root canal and compressed with a #70 K-file (Figure 4A). Immediately after, the MTA cement was inserted into the apical third of the canal (Figure 4B). Having established the formation of the apical barrier, including filling the foraminal area enlargement, root canal was filled with gutta-percha and sealer (AH Plus; DeTrey Dentsply GmbH, Konstanz, Germany) using lateral condensation technique (Figure 4C).

Upon completion of endodontic treatment, coronal access was temporarily restored with glass ionomer cement (Vidreon; SS White, Rio de Janeiro, RJ, BR) and the patient was oriented to follow the definitive restorative procedures (Figure 4D). A radiographic control was taken 6 months later, showing increase of bone density around the root. There were no reports of clinical signs and symptoms and the tooth was properly restored and maintained in normal function in the oral cavity.

## Discussion

In the presented cases placement of an apical barrier with MTA allowed the treatment of open apex teeth in a single treatment session, avoiding the risk of root canal contamination and/or radicular fracture, as well as optimizing the time of endodontic treatment [7, 8, 18, 19]. Although the satisfactory physicochemical and biological properties of the MTA cement

are well-recognized [2], the use of the material should be restricted to root canal [2, 20].

Thus, the use of an apical barrier with a biocompatible material, before MTA placement is an interesting treatment strategy for avoiding extrusion of the material beyond the radicular apex [13, 14]. The collagen membrane is used in periodontal guided tissue regeneration due to its excellent biological properties and being resorbable [21]. Nevertheless, the amount to be used, the material cost and the difficulty of handling are amongst the main disadvantages of collagen membrane [13, 21]. 

Moreover, the lyophilized collagen hemostatic sponge is easy to handle and its tissue tolerability is satisfactory, since its insertion in the apical radicular third can be performed with the aid of a specific gutta-percha condenser or endodontic file [22-24]. The lyophilized collagen sponge used in presented cases, is absorbable and has porcine origin, with better biocompatibility than that obtained from animal skin (Gelfoam), because in 24 days it promotes complete alveolar bone healing with presence of trabecular bone and large amount of blood vessels and fibroblasts [19]. Possibly, this healing process also occurs after placement of the lyophilized collagen sponge in the radicular apical third in open apex cases, since healing of the periapical lesion is similar to alveolar bone socket [25, 26].

After apical condensation of lyophilized collagen, clinically it is possible to confirm the presence of apical barrier by using an endodontic file. This allowed the safe placement of the MTA apical barrier, with approximately 4 mm thickness. Acute inflammatory reaction in the alveolar bone socket subsides after 5 days of the implantation of lyophilized collagen sponge [19]. Therefore, there was time for the hydration process and setting of the MTA cement to occur, preventing its interference on periradicular healing process [27, 28].

MTA cement has osteogenic activity and good bone tissue tolerance [29-31]. However, due to its high alkalinity a risk of necrosis exists if there is direct contact with the apical tissues, for instance after accidental apical overfilling [32, 33]. Therefore, the apical barrier with lyophilized collagen sponge and MTA cement allows the root canal filling in single session, with safety and non-invasive procedures.

## Conclusion

These reports showed that treatment of open-apex teeth with placement of an apical matrix with lyophilized collagen sponge against which MTA apical plug can be condensed, has favorable prognosis.

## References

[1] Love RM, Firth N (2009). Histopathological profile of surgically removed persistent periapical radiolucent lesions of endodontic origin. Int Endod J.

[2] Parirokh M, Torabinejad M (2010). Mineral trioxide aggregate: a comprehensive literature review--Part III: Clinical applications, drawbacks, and mechanism of action. J Endod.

[3] Trope M (2010). Treatment of the immature tooth with a non-vital pulp and apical periodontitis. Dent Clin North Am.

[4] Tomson RM, Polycarpou N, Tomson PL (2014). Contemporary obturation of the root canal system. Br Dent J.

[5] Chutich MJ, Kaminski EJ, Miller DA, Lautenschlager EP (1998). Risk assessment of the toxicity of solvents of gutta-percha used in endodontic retreatment. J Endod.

[6] Andreasen JO, Munksgaard EC, Bakland LK (2006). Comparison of fracture resistance in root canals of immature sheep teeth after filling with calcium hydroxide or MTA. Dent Traumatol.

[7] Yassen GH, Platt JA (2013). The effect of nonsetting calcium hydroxide on root fracture and mechanical properties of radicular dentine: a systematic review. Int Endod J.

[8] Verissimo RD, Gurgel-Filho ED, De-Deus G, Coutinho-Filho T, de Souza-Filho FJ (2010). Coronal leakage of four intracanal medications after exposure to human saliva in the presence of a temporary filling material. Indian J Dent Res.

[9] Binnie WH, Rowe AH (1973). A histological study of the periapical tissues of incompletely formed pulpless teeth filled with calcium hydroxide. J Dent Res.

[10] Roberts SC Jr, Brilliant JD (1975). Tricalcium phosphate as an adjunct to apical closure in pulpless permanent teeth. J Endod.

[11] Rossmeisl R, Reader A, Melfi R, Marquard J (1982). A study of freeze-dried (lyophilized) cortical bone used as an apical barrier in adult monkey teeth. J Endod.

[12] Rossmeisl R, Reader A, Melfi R, Marquard J (1982). A study of freeze-dried (lyophilized) dentin used as an apical barrier in adult monkey teeth. Oral Surg Oral Med Oral Pathol.

[13] Gharechahi M, Ghoddusi J (2012). A nonsurgical endodontic treatment in open-apex and immature teeth affected by dens invaginatus: using a collagen membrane as an apical barrier. J Am Dent Assoc.

[14] Nayak G, Hasan MF (2014). Biodentine-a novel dentinal substitute for single visit apexification. Restor Dent Endod.

[15] Moore A, Howley MF, O'Connell AC (2011). Treatment of open apex teeth using two types of white mineral trioxide aggregate after initial dressing with calcium hydroxide in children. Dent Traumatol.

[16] Floratos SG, Tsatsoulis IN, Kontakiotis EG (2013). Apical barrier formation after incomplete orthograde MTA apical plug placement in teeth with open apex--report of two cases. Braz Dent J.

[17] Benoit PW, Hunt LM (1976). Comparison of a microcrystalline collagen preparation and gelatin foam in extraction wounds. J Oral Surg.

[18] Rudagi KB, Rudagi B (2012). One-step apexification in immature tooth using grey mineral trioxide aggregate as an apical barrier and autologus platelet rich fibrin membrane as an internal matrix. J Conserv Dent.

[19] IA B, LA M, T O, R O, J N (2006). Implantes das esponjas hemostáticas Gelfoam® e Hemospon® em alvéolos dentais, em ratos, após exodontia Estudo histológico comparativo. Rev Cien Odont.

[20] Tahan E, Celik D, Er K, Tasdemir T (2010). Effect of unintentionally extruded mineral trioxide aggregate in treatment of tooth with periradicular lesion: a case report. J Endod.

[21] Matsumoto G, Hoshino J, Kinoshita Y, Sugita Y, Kubo K, Maeda H, Ikada Y, Kinoshita Y (2012). Alveolar bone regeneration using poly-(lactic acid-co-glycolic acid-co-epsilon-caprolactone) porous membrane with collagen sponge containing basic fibroblast growth factor: an experimental study in the dog. J Biomater Appl.

[22] Coomes AM, Mealey BL, Huynh-Ba G, Barboza-Arguello C, Moore WS, Cochran DL (2014). Buccal bone formation after flapless extraction: a randomized, controlled clinical trial comparing recombinant human bone morphogenetic protein 2/absorbable collagen carrier and collagen sponge alone. J Periodontol.

[23] Shimoji S, Miyaji H, Sugaya T, Tsuji H, Hongo T, Nakatsuka M, Uz Zaman K, Kawanami M (2009). Bone perforation and placement of collagen sponge facilitate bone augmentation. J Periodontol.

[24] Sood R, Kumar Hans M, Shetty S (2012). Apical barrier technique with mineral trioxide aggregate using internal matrix: a case report. Compend Contin Educ Dent.

[25] Bystrom A, Happonen RP, Sjogren U, Sundqvist G (1987). Healing of periapical lesions of pulpless teeth after endodontic treatment with controlled asepsis. Endod Dent Traumatol.

[26] Okamoto T, Alves-Rezende MC, Claudio CC, Rodrigues Tda S, Okamoto R (2006). Effects of Tissucol and epsilon aminocaproic acid in the healing process following dental extraction in dehydrated rats. Braz Oral Res.

[27] Danesh F, Tootian Z, Jahanbani J, Rabiee M, Fazelipour S, Taghva O, Shabaninia S (2010). Biocompatibility and mineralization activity of fresh or set white mineral trioxide aggregate, biomimetic carbonated apatite, and synthetic hydroxyapatite. J Endod.

[28] Lee BN, Kim HJ, Chang HS, Hwang IN, Oh WM, Kim JW, Koh JT, Min KS, Choi CH, Hwang YC (2014). Effects of mineral trioxide aggregate mixed with hydration accelerators on osteoblastic differentiation. J Endod.

[29] Lee BN, Lee KN, Koh JT, Min KS, Chang HS, Hwang IN, Hwang YC, Oh WM (2014). Effects of 3 endodontic bioactive cements on osteogenic differentiation in mesenchymal stem cells. J Endod.

[30] Rahimi S, Mokhtari H, Shahi S, Kazemi A, Asgary S, Eghbal MJ, Mesgariabbasi M, Mohajeri D (2012). Osseous reaction to implantation of two endodontic cements: Mineral trioxide aggregate (MTA) and calcium enriched mixture (CEM). Med Oral Patol Oral Cir Bucal.

[31] Gomes-Filho JE, de Moraes Costa MT, Cintra LT, Lodi CS, Duarte PC, Okamoto R, Bernabe PF, Nery MJ, Cannon M (2010). Evaluation of alveolar socket response to Angelus MTA and experimental light-cure MTA. Oral Surg Oral Med Oral Pathol Oral Radiol Endod.

[32] Weckwerth PH, Machado AC, Kuga MC, Vivan RR, Polleto Rda S, Duarte MA (2012). Influence of radiopacifying agents on the solubility, pH and antimicrobial activity of portland cement. Braz Dent J.

[33] Chavez-Andrade GM, Kuga MC, Duarte MA, Leonardo Rde T, Keine KC, Sant'Anna-Junior A, So MV (2013). Evaluation of the physicochemical properties and push-out bond strength of MTA-based root canal cement. J Contemp Dent Pract.

